# EphrinB2 overexpression enhances osteogenic differentiation of dental pulp stem cells partially through ephrinB2-mediated reverse signaling

**DOI:** 10.1186/s13287-019-1540-2

**Published:** 2020-01-29

**Authors:** Wen Wang, Changyong Yuan, Tengyu Geng, Yi Liu, Shaoyue Zhu, Chengfei Zhang, Zongxiang Liu, Penglai Wang

**Affiliations:** 1 Affiliated Stomatological Hospital of Xuzhou Medical University, No. 130 Huaihai West Road, Xuzhou, 221000 Jiangsu China; 2 Faculty of Dentistry, The University of Hong Kong, Pokfulam, Hong Kong, 999077 China

**Keywords:** EphrinB2, Osteogenesis, Bone regeneration, DPSCs

## Abstract

**Background:**

Alveolar bone loss is a frequent occurrence. Dental pulp stem cells (DPSCs) which have invasive accessibility and high osteogenic potential is a promising source for cell-based bone regeneration. EphrinB2 is involved in bone homeostasis and osteogenesis. The aim of this study was to investigate the effect and mechanism of ephrinB2 overexpression on osteogenic differentiation of DPSCs and bone defect repair.

**Methods:**

EphrinB2 expression was analyzed during osteogenic induction of human DPSCs (hDPSCs). Endogenous ephrinB2 expression in hDPSCs was then upregulated using EfnB2 lentiviral vectors. The effect of ephrinB2 overexpression on osteogenic differentiation capacity of hDPSCs was investigated in vitro, and activation of ephrinB2-EphB4 bidirectional signaling in ephrinB2-overexpressing hDPSCs was detected. In vivo, a canine alveolar bone defect model was established and canine DPSCs (cDPSCs) were cultured, characterized, EfnB2-tranfected, and combined with a PuraMatrix scaffold. Micro-CT analysis was performed to evaluate the therapeutic effect of ephrinB2-overexpressing cDPSCs on bone defect repair.

**Results:**

EphrinB2 was upregulated after osteogenic induction of hDPSCs. EphrinB2 overexpression enhanced osteogenic differentiation capacity of hDPSCs in vitro. Moreover, p-ephrinB2 instead of p-EphB4 was upregulated by ephrinB2 overexpression, and activation of ephrinB2-mediated reverse signaling promoted osteogenic differentiation of hDPSCs. In a canine bone defect model, ephrinB2 overexpression in cDPSCs significantly improved trabecular bone volume per tissue volume (BV/TV) and trabecular thickness, as demonstrated by radiographic analysis.

**Conclusions:**

EphrinB2 overexpression enhanced osteogenic potential of DPSCs partially via upregulation of ephrinB2-mediated reverse signaling and effectively promoted alveolar bone defect repair.

**Electronic supplementary material:**

The online version of this article (10.1186/s13287-019-1540-2) contains supplementary material, which is available to authorized users.

## Background

Bone loss is one of the most challenging issues in dentistry. Clinically, various conditions such as inflammation, trauma, congenital malformation, and cancer can cause oral maxillofacial bone defects [1], and some systematic factors such as aging [2, 3], osteoporosis [4], and diabetes [5] may aggravate bone loss and disrupt bone repair. Bone loss and consequent tooth loss severely affect chewing, pronunciation, esthetics, and mental health, so bone regeneration is of primary concern. Cell-based therapy and bone tissue engineering are now becoming potentially promising strategies [6–8].

Dental pulp stem cells (DPSCs) are characterized as mesenchymal stem cells (MSCs), with high levels of self-renewal and proliferation, a characteristic immunophenotype and potential of multiple differentiation [9–11]. Compared with bone MSCs (BMSCs), DPSCs can be easily and invasively obtained from the discarded or removed teeth, which makes DPSCs a promising source for autologous cell therapy. Although the natural function of DPSCs is to differentiate into odontoblasts producing reparative dentin [12], growing evidence has proved the ability of DPSCs for generating bone-like tissues and repairing bone loss [13–17]. The osteogenic differentiation capability of DPSCs is crucial for their application in bone regeneration and has been well documented by several studies [18–21]. In addition, it has been reported that DPSCs exhibit higher osteogenic potential along with decreased adipogenic potential compared to BMSCs [22].

Ephrin is the ligand for the tyrosine kinase receptor Eph, and the ephrin/Eph interaction plays a key role in many biological processes such as angiogenesis [23], axon guidance [24], cell migration [25, 26], and skeletal patterning [27]. Ephrin ligands are divided into ephrinA ligands (A1-A5), which are membrane-anchored proteins, and ephrinB ligands (B1-B3), which are transmembrane proteins. In general, ephrinB ligands preferentially bind to EphB receptors, with a few exceptions [28, 29]. The ephrinB/EphB interaction leads to activation of bidirectional signaling: the forward signaling is mediated by EphB receptors and the reverse signaling is mediated by ephrinB ligands. Both forward and reverse signaling can activate downstream signaling cascades and regulate biological processes.

EphrinB2 is one of three ephrinB ligands, and its involvement in bone homeostasis and osteogenesis was firstly verified by Zhao et al. [30]. They found that ephrinB2 expressed on osteoclasts could stimulate EphB4-mediated forward signaling in osteoblasts, resulting in enhanced osteoblast differentiation [30]. Similarly, exogenous recombinant ephrinB2-Fc has been demonstrated to promote osteoblast differentiation [31, 32]. In fact, ephrinB2 and their receptors are co-expressed on osteoblasts [30], and the important role of endogenous ephrinB2 expressed on osteoblasts for regulating differentiation and promoting bone mineralization has also been shown [33, 34]. Excluding osteoblasts, ephrinB2 and its receptors are also co-expressed in BMSCs [35] and DPSCs [36]. Previous studies have reported that exogenous ephrinB2-Fc treatment can upregulate osteogenic gene expression and promote mineralized nodule formation in BMSCs [37–39] and DPSCs [36], while the role of endogenous ephrinB2 is unclear.

In this study, we aimed to investigate the effect of endogenous ephrinB2 overexpression on osteogenic differentiation of DPSCs. We observed that ephrinB2 overexpression enhanced osteogenic differentiation of DPSCs partially through ephrinB2-mediated reverse signaling. Furthermore, we found that transplantation of ephrinB2-overexpressing DPSCs promoted repairing alveolar defect in a beagle model.

## Methods

### Ethics statement

Collection of human third molars was carried out after receiving approval from the Ethics Committee of Xuzhou Medical University (20161108) and written informed consent from volunteers. The dog experiments in this study were approved by the Experimental Animal Ethics Committee of Xuzhou Medical University (20161108). All experimental procedures were carried out in accordance with relevant guidelines and regulations. Dogs were looked after by professional breeders, and no cases of animal cruelty occurred.

### Primary cell culture and identification

Human DPSCs (hDPSCs) were obtained from extracted third molars of healthy volunteers (18–25 years old), and canine DPSCs (cDPSCs) were obtained from sound anterior teeth of six beagles (15-month-old; 10–15 kg; male). Briefly, pulp tissues were isolated from pulp cavities, washed several times, minced, and digested with 3 mg/ml collagenase type I (Gibco, Grand Island, NY, USA) and 4 mg/ml dispase (Gibco, Grand Island, NY, USA) for 1 h at 37 °C. The human pulp samples from diverse individuals were mixed and pooled to decrease individual differences, while each dog’s DPSCs were cultured separately to avoid the immunological rejection after transplantation. After digestion, cells and remaining tissues were suspended in growth medium containing α-minimum essential medium (α-MEM; Gibco, Beijing, China), 20% fetal bovine serum (FBS; Gibco, South America), 100 U/ml penicillin, and 100 μg/ml streptomycin (Vicmed, Xuzhou, Jiangsu, China) at 37 °C and 5% CO_2_. Cells below passage six were used in experiments.

Self-renewal ability was confirmed by a colony-formation assay. Briefly, 400 cells were dispersedly seeded in a 6-cm dish and cultured for 10 days. Then, cell colonies were fixed and stained with crystal violet.

Multiple differentiation of hDPSCs and cDPSCs were examined. To induce osteogenic differentiation, 10 mmol/L β-glycerophosphate, 50 μg/ml L-ascorbic acid phosphate, and 10 nmol/L dexamethasone were added to the growth medium. To induce adipogenic differentiation, 1 μmol/L dexamethasone, 1 μg/ml insulin, and 0.5 mmol/L 3-isobutyl-1-methylxanthin were supplemented to the growth medium. To induce neurogenic differentiation, cells were cultured in Neurobasal A medium (Gibco-Thermo Fisher Scientific, Grand Island, NY, USA) supplemented with 40 ng/ml basic fibroblast growth factor (bFGF; PeproTech, Rocky Hill, NJ, USA) and 20 ng/ml epidermal growth factor (EGF; PeproTech, Rocky Hill, NJ, USA). Four weeks later, Alizarin Red S staining, Oil Red O staining, and immunofluorescence staining for β III–tubulin were carried out, respectively.

In addition, flow cytometry and immunofluorescence were performed to detect mesenchymal stem cell markers for human and canine DPSCs, respectively. The following fluorescent conjugated anti-human antibodies were used for flow cytometry: CD90 PerCP, CD73 FITC, CD45 APC, CD105 APC (BD Biosciences, San Jose, CA, USA), and STRO-1 PE (Santa Cruz Biotechnology, Santa Cruz, CA, USA). For immunofluorescence, the fixed cDPSCs were blocked with 5% bovine serum albumin and incubated overnight at 4 °C with primary monoclonal antibodies for CD73, CD90, CD105, CD45 (1:100, Affinity Biosciences, Changzhou, Jiangsu, China), or STRO-1 (R&D Systems, Wiesbaden, Germany). And then, fluorescein-conjugated goat anti-rabbit IgG(H+L) antibody or NL493-conjugated goat anti-mouse IgM antibody were used.

### Cell transfection

EfnB2 lentiviral particles for human (LPP-M0409-Lv233-400) and dog (LPP-GS-Md02143-Lv201-400) and corresponding EGFP lentiviral particles (LPP-EGFP-Lv233-100 for human; LPP-NEG-Lv201–100 for dog) were purchased from GeneCopoeia (Rockville, Maryland, USA). hDPSCs and cDPSCs (3 × 10^5^cells/well) at passage one were seeded in six-well plates. When cell confluence reached 70%, cells were incubated with EfnB2 lentiviral particles (40 μl/well) or control lentiviral particles (8 μl/ well) along with 4 μg/ml polybrene in growth medium for 12 h. Three days later, transfected cells were selected by 1.5 μg/ml puromycin and passaged for subsequent use. Transfection efficiency was verified by green fluorescence expression and ephrinB2 upregulation.

### Cell proliferation assay

To assess the influence of EfnB2 transfection on proliferation of hDPSCs, a cell proliferation assay was performed. Related cells (5000 cells/well) were planted into 96-well plates. On days 0, 2, 4, 6, and 8, the medium in each well was changed with 100 μl α-MEM supplemented with 10% Cell Counting Kit-8 (CCK-8; Vicmed, Xuzhou, Jiangsu, China). One hour later, absorbance at 450 nm was measured.

### Cell migration assay

To assess the influence of EfnB2 transfection on migration capability of hDPSCs, related cells were plated in the upper chamber of a transwell-24-well permeable support with 0.8-μm pore polyester membrane (Corning, NY, USA) at a density of 5 × 10^4^ cells per well. α-MEM was added into the upper chamber, while α-MEM supplemented with 10% FBS was in the lower chamber. The cells migrating to the lower surface of membrane were fixed, stained, and counted after 6 and 9 h.

### Osteogenic induction

To investigate the osteogenic differentiation of hDPSCs and cDPSCs under diverse treatment, related cells were plated in six-well plates (3 × 10^5^ cells/well) and induced in osteogenic medium. Extracellular mineralized nodules were stained by 2% (*w*/*v*) Alizarin Red S solution (pH 4.2) for 30 min. Alkaline phosphatase (ALP) staining was tested by nitro-blue tetrazolium/5-bromo-4-chloro-3′-indolyphosphate (NBT/BCIP) substrate solution (Beyotime, Shanghai, China) for 1 h. Intensities of Alizarin Red S staining and ALP staining were quantified with ImageJ (Rawak Software, Germany). Osteogenic gene transcription was analyzed by quantitative real-time reverse transcription-polymerase chain reaction (qRT-PCR).

### Stimulation with EphB4-Fc

Recombinant EphB4-Fc chimeras (R&D Systems, Wiesbaden, Germany) were used to stimulate ephrinB2-mediated reverse signaling, and IgG-Fc (R&D Systems, Wiesbaden, Germany) was used as the negative control. hDPSCs were cultured in osteogenic medium supplemented with 2 or 4 μg/ml EphB4-Fc or 4 μg/ml IgG-Fc. Alizarin Red S staining was performed on day 14, and osteogenic gene transcription was measured on day 7.

### Quantitative reverse transcription-polymerase chain reaction (qRT-PCR)

Total RNA was isolated using TRIzol (Invitrogen, Carlsbad, CA, USA) according to the manufacturer’s protocol and 1 μg RNA was reverse transcribed into cDNA by HiScript Q RT SuperMix for qRT-PCR (Vazyme, Nanjing, Jiangsu, China) in a 20 μl reaction volume. qRT-PCR was performed with UltraSYBR Mixture (Cwbio, Beijing, China) on an ABI7500 quantitative PCR instrument (Applied Biosystems, Darmstadt, Germany). A total of 20 μl reaction system was used: 2 μl cDNA, 100 nM forward and reverse primers, and 10 μl 1× UltraSYBR Mixture. Primers are listed in Table 1. The cycle conditions were as follows: initial denaturation at 95 °C for 10 min, 40-cycle amplification of 95 °C for 15 s and 60 °C for 1 min, and melt curve analysis of 95 °C for 15 s, 60 °C for 1 min, 95 °C for 15 s, and 60 °C for 15 s. Relative expression was calculated by the comparative cycle threshold method (ΔΔCT) and normalized to β-actin.

**Table 1:** The sequences of canine and human primers used in qRT-PCR

Species	Gene	Primers
*Homo sapiens*	ephrinB2	For: 5′-TATGCAGAACTGCGATTTCCAA-3′ Rev: 5′-TGGGTATAGTACCAGTCCTTGTC-3′
	ALP	For: 5′-CCTCGTTGACACCTGGAAGAG-3′ Rev: 5′-TTCCGTGCGGTTCCAGA-3′
	RUNX2	For: 5′-TCTTAGAACAAATTCTGCCCTTT-3′ Rev: 5′-TGCTTTGGTCTTGAAATCACA-3′
	BMP2	For: 5′-TTCCACCATGAAGAATCTTTGGA-3′ Rev: 5′-CCTGAAGCTCTGCTGAGGTGAT-3′
	COL1	For: 5′-GAGGGCCAAGACGAAGACATC-3′ Rev:5′- CAGATCACGTCATCGCACAAC-3′
	OCN	For: 5′-CTACCTGTATCAATGGCTGGG-3′ Rev: 5′-GGATTGAGCTCACACACCT-3′
	DSPP	For: 5′-TTTGGGCAGTAGCATGGGC-3′ Rev: 5′-CCATCTTGGGTATTCTCTTGCCT-3′
	DMP1	For: 5′-CTCCGAGTTGGACGATGAGG-3′ Rev: 5′-TCATGCCTGCACTGTTCATTC-3′
	EphB4	For: 5′-CGCACCTACGAAGTGTGTGA-3′ Rev: 5′-GTCCGCATCGCTCTCATAGTA-3′
	β-actin	For: 5′-ACGTTGCTATCCAGGCTGTG-3′ Rev: 5′-GGCCATCTCTTGCTCGAAGT-3′
*Canis familiaris*	RUNX2	For: 5′-TACCACACCTACCTGCCACCAC-3′ Rev: 5′-GCGGAAGCATTCTGGAAGGAGAC-3′
	BMP2	For: 5′-TGAACTCCACTAACCACGCCATTG-3′ Rev: 5′-TGTTGGTACACAGCACGCCTTG-3′
	ephrinB2	For: 5′-TGCCAGACAAGAGCCATGAAGATC-3′ Rev: 5′-GGCGTCGTGTTGGATCATTATGC-3′
	β-actin	For: 5′-ATCACTATTGGCAACGAGCGGTTC-3′ Rev: 5′-CAGCACTGTGTTGGCATAGAGGTC-3′

*Abbreviations*: *ALP* alkaline phosphatase, *RUNX2* runt-related transcription factor 2, *BMP2* bone morphogenetic protein 2, *COL1* collagen type I, *OCN* osteocalcin, *DSPP* dentin sialophosphoprotein, *DMP1* dentin matrix protein 1, *For* forward, *Rev* reverse

### Western blot analysis

Cells were lysed with lysis buffer (50 mM Tris [pH 7.4], 150 mM NaCl, 1% Triton X-100, 1% sodium deoxycholate, 0.1% SDS, sodium orthovanadate, sodium fluoride, EDTA, leupeptin and 1 mM phenylmethanesulfonyl fluoride) (Beyotime, Shanghai, China). Forty micrograms of total protein underwent 10% sodium dodecyl sulfate polyacrylamide gel electrophoresis and was subsequently transferred to nitrocellulose membranes (Pall Corporation, Pensacola, Florida, USA). The membranes were blocked with 5% skim milk (Vicmed, Xuzhou, Jiangsu, China) for 1 h at room temperature and incubated with primary antibodies for ephrinB2 (1:2000, clone number EPR10072(B), Abcam, Cambridge, UK), phospho-ephrinB2 (Tyr324/329, 1:500, Cell Signaling Technology, Danvers, MA, USA), EphB4 (1:200; Santa Cruz Biotechnology, Dallas, TX, USA), phospho-EphB4 (1:1000; Signalway Antibody, College Park, MD, USA), EphB1 (1:50; Affinity Biosciences, Changzhou, Jiangsu, China), EphB2 (1:50; Affinity Biosciences, Changzhou, Jiangsu, China), or β-actin (1:3000, Beyotime, Shanghai, China) overnight at 4 °C. After washing with PBST, the membranes were incubated with horseradish peroxidase-conjugated secondary antibodies (Proteintech, Wuhan, Hubei, China) for 2 h at room temperature. Protein blots were detected using a chemiluminescence kit (NCM Biotech, Suzhou, Jiangsu, China) and Tanon 4500 Immunodetection System (Tanon, Shanghai, China). Gray values were analyzed by ImageJ (Rawak Software, Germany).

### Endogenous RhoA activity assay

Active GTP-RhoA was captured using the RhoA Pull-down Activation Assay Biochem Kit (bead pull-down format) (Cytoskeleton, Inc., Japan). Briefly, cell lysates were incubated with GST-rhotekin-RBD beads for 1 h at 4 °C. The protein/beads complexes were washed and the bound proteins were resuspended. GTP-RhoA and total RhoA were detected by western blotting with a RhoA-specific antibody.

### Cell growth in PuraMatrix

To deliver cells into defect areas, cells were encapsulated in PuraMatrix Peptide Hydrogel (Corning, Bedford, USA). PuraMatrix is a type I self-assembling peptide (SAPs), which can self-assemble into a 3D structured hydrogel under certain physiological conditions. The proliferation of cDPSCs in 0.5%, 0.25%, or 0.125% PuraMatrix was measured. cDPSCs were suspended in varying dilutions of PuraMatrix in sucrose and added into 96-well plates. Then gelation was induced by careful addition of 100 μl growth medium onto the gel. Medium was changed twice in the next 1 h to equilibrate pH. On days 1, 3, 5, and 7, 10 μl CCK-8 regent was added into 100 μl growth medium and absorbance at 450 nm was measured 1 h later. To assess proliferation of cDPSCs at different densities in 0.25% PuraMatrix, cDPSCs (0.25, 0.5, 1, 2 or 4 × 10^6^ cells/ml) encapsulated with 0.25% PuraMatrix were seeded into 96-well plates. Cell growth was measured on days 1, 3, 5, and 7.

### Alveolar bone defect model establishment and cell transplantation

The six beagle dogs whose cDPSCs had been isolated before were used to establish bone defect models. All surgical procedures were performed under general anesthesia, which was induced with propofol (5–7 mg/kg, i.v.) and maintained by isoflurane inhalation (1.5–2% isoflurane/O_2_ to effect). Three months after extraction of bilateral mandibular third premolars, horizontal incisions were made between the second and fourth premolars, and mucoperiosteal flaps were elevated. Bilateral four-wall critical-sized alveolar bone defects (4 × 2 × 5 mm, length × width × depth) were created mesial to the fourth premolars and distal to the second premolars with a 1–2-mm distance between defects and premolars. There were four defects for each dog, which were randomly assigned into four groups (*n* = 3 per group per time point): NC group (without any treatment), PuraMatrix group, Vector-cDPSCs + PuraMatrix group, and EfnB2-cDPSCs + PuraMatrix group. For the latter two groups, cells encapsulated with PuraMatrix were cultured in osteogenic medium for 7 days before transplantation in vivo. The wounds were sutured without stress. At the 4th and 8th week after the surgery, the mandibles were fixed by arterial perfusion of 10% formalin and the dogs were euthanized by an overdose of anesthetic. The bone segments containing defect areas were separated, fixed, and scanned by micro-CT.

### Micro-CT analysis

For micro-CT evaluation, samples were scanned by a micro-CT scanner (Scanco Medical AG, Brüttisellen, Zurich, Switzerland) at 80 kV, 116 μA. The slice thickness was 25 μm. TRI/3D-BON (Ratoc System Engineering, Tokyo, Japan) was used to perform 3-D structural analysis. The original defect areas, whose borders were visually recognizable, were defined as the region of interest. Trabecular bone volume per tissue volume (BV/TV), trabecular number (Tb.N), trabecular thickness (Tb.Th), trabecular spacing (Tb.Sp), connectivity density (Conn-Den), and structure model index (SMI) were measured.

### Statistical analysis

All experiments were repeated at least in triplicate. Collected data were analyzed using SPSS 19.0 (IBM Corp, Armonk, NY, USA), and results were displayed as mean ± standard deviation. Comparison between two groups was analyzed with two-tailed Student’s *t* test and differences among more than two groups were determined by a one-way ANOVA followed by Bonferroni’s post hoc test. A value of *p* < 0.05 was accepted as statistically significant.

## Results

### Characterization of primary cultured hDPSCs

**Fig. 1:**
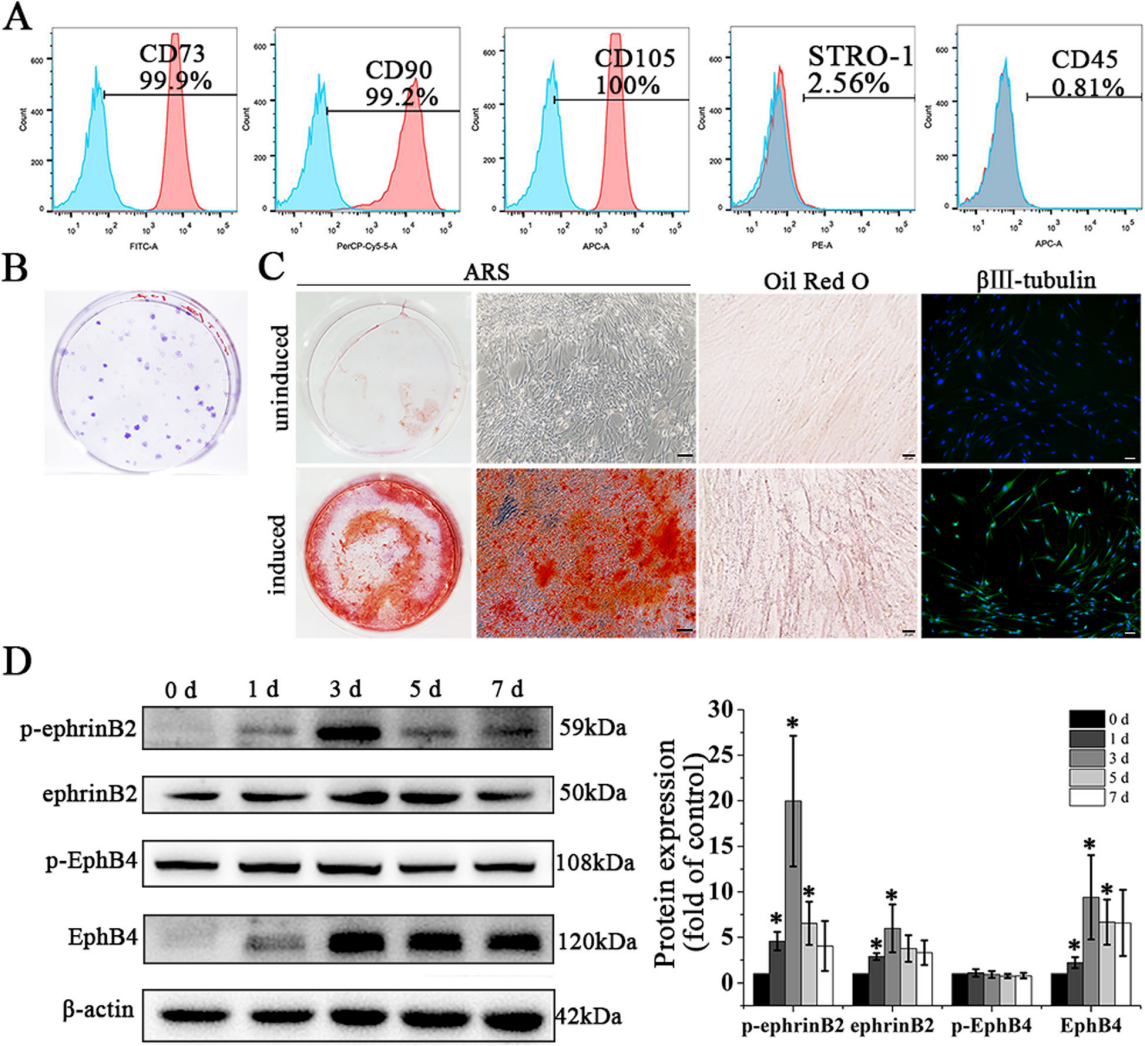
Characterization of primary cultured hDPSCs and ephrinB2 expression in hDPSCs during osteogenic differentiation. **a** Mesenchymal stem cell markers measured by flow cytometry. **b** Colony-forming units stained with crystal violet. **c** Osteogenic, adipogenic, and neurogenic potentials of hDPSCs were confirmed by Alizarin Red S staining, Oil Red O staining, and β III -tubulin expression. Scale bar of left and right images, 100 μm; scale bar of middle images, 20 μm. **d** Expression of p-ephrinB2, ephrinB2, p-EphB4, and EphB4 in hDPSCs during osteogenic differentiation. Protein expression levels were normalized to that of β-actin. Data are shown as mean ± SD. Assays were repeated three times. *p < 0.05 vs. sample on day 0 of osteogenic induction

Flow cytometry analysis of stem cell surface markers showed that hDPSCs were positive for CD73 (99.9%), CD90 (99.2%), and CD105 (100%), and negative for CD45 (0.81%). Also, 2.56% of hPDLSCs were positive for STRO-1 (Fig. 1a). Cell colonies were observed after 10 days of culture (Fig. 1b). Osteogenic, adipogenic, and neurogenic differentiation of hDPSCs were confirmed by mineralized nodule formation, lipid-rich vacuole accumulation and β III-tubulin expression, respectively (Fig. 1c).

### EphrinB2 expression in hDPSCs during osteogenic induction

We investigated ephrinB2 expression level during osteogenic induction of hDPSCs. Results showed that ephrinB2 and its phosphorylated form (p-ephrinB2) were upregulated on days 1, 3, and 5 of induction and on days 1 and 3 of induction, respectively (Fig. 1d). EphB4 is a receptor of ephrinB2, and the EphB4-ephrinB2 interaction specifically stimulates osteogenesis, so we measured EphB4 and p-EphB4 expression simultaneously. EphB4 was upregulated on days 1, 3, and 5 of osteogenic induction compared to day 0, while p-EphB4 expression showed no significant change during osteogenic induction of hDPSCs (Fig. 1d).

### Recombinant EfnB2-lentivirus transfection and its influence on proliferation and migration of hDPSCs

**Fig. 2:**
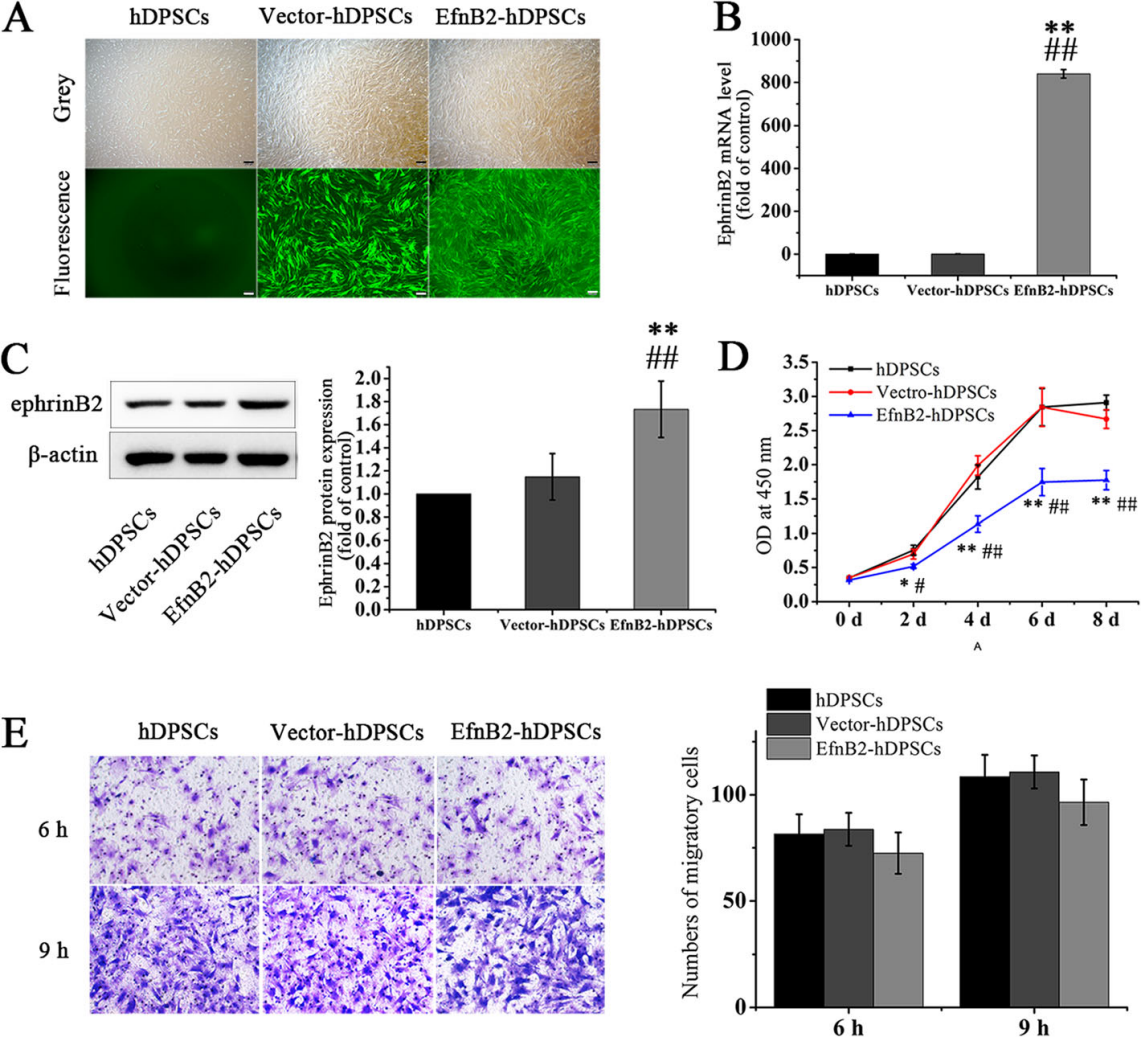
Establishment of ephrinB2-overexpressing hDPSCs and the influence of ephrinB2 overexpression on proliferation and migration. **a** Green fluorescence was observed in lentivirus-infected hDPSCs. Scale bar = 200 μm. **b, c** Verification of ephrinB2 overexpression in hDPSCs. **d** Cell proliferation was examined by CCK-8 assay. **e** Cells migrating to the lower surface of membranes were stained and counted at the 6th and 9th hour. Data are shown as mean ± SD. Assays were repeated three times. *p < 0.05 and **p < 0.01 vs. hDPSCs; #*p* < 0.05 and ##*p* < 0.01 vs. Vector-hDPSCs

EphrinB2-Fc has been reported to stimulate osteogenic differentiation of hDPSCs, while the influence of endogenous ephrinB2 overexpression on biological functions of hDPSCs is unclear. To clarify this issue, we constructed ephrinB2-overexpressing hDPSCs (EfnB2-hDPSCs) via recombinant EfnB2-lentivirus transfection, and hDPSCs infected with corresponding control lentivirus were treated as a control (Vector-hDPSCs). Three days after infection, green fluorescence was visible in both EfnB2-hDPSCs and Vector-hDPSCs (Fig. 2a). In comparison with uninfected hDPSCs and Vector-hDPSCs, ephrinB2 mRNA and protein were significantly upregulated in EfnB2-hDPSCs (Fig. 2b, c), which indicated successful establishment of ephrinB2- overexpressed hDPSCs. The consequence of EfnB2 transfection on proliferation and migration of hDPSCs were then analyzed. Results showed that EfnB2-hDPSCs proliferated more slowly than uninfected hDPSCs and Vector-hDPSCs (Fig. 2d), while there was no significant difference in migration capability among uninfected hDPSCs, Vector-hDPSCs, and EfnB2-hDPSCs (Fig. 2e).

### EphrinB2 overexpression enhanced calcium deposition and increased ALP expression and osteogenic gene transcription

**Fig. 3:**
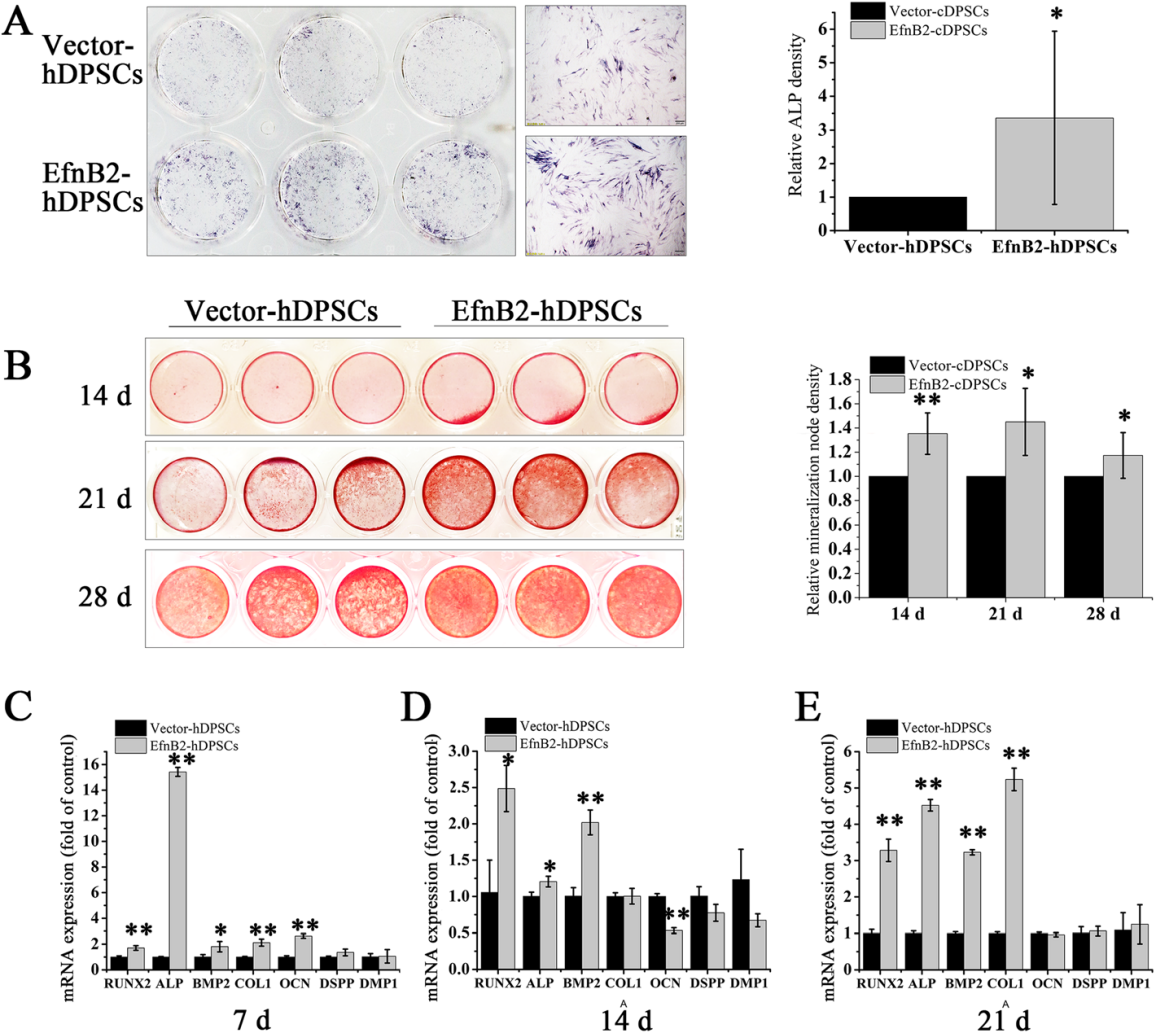
The effect of endogenous ephrinB2 overexpression on osteogenic differentiation of hDPSCs. **a** ALP staining on day 7 of osteogenic differentiation. Scale bar = 200 μm. Staining intensity was quantified with ImageJ. **b** Alizarin Red S staining on days 14, 21, and 28 of osteogenesis. Staining intensity was quantified with ImageJ. **c**–**e** mRNA expression of RUNX2, ALP, BMP2, COL1, OCN, DSPP, and DMP1 in EfnB2-hDPSCs (ephrinB2 overexpression) and Vector-hDPSCs (ephrinB2 overexpression control) after 7, 14, and 21 days of osteogenic induction. Data are shown as mean ± SD. Assays were repeated three times. *p < 0.05 and **p < 0.01 vs. Vector-hDPSCs

To compare the osteogenic potential between EfnB2-hDPSCs and Vector-hDPSCs, ALP staining, Alizarin Red S staining and analysis of osteogenic gene transcription were performed. On day 7, ALP expression was upregulated in EfnB2-hDPSCs compared to Vector-hDPSCs (Fig. 3a). Calcium deposits in EfnB2-hDPSCs were significantly more abundant than that in Vector-hDPSCs on days 14, 21, and 28 of induction (Fig. 3b). qRT-PCR analysis showed that runt-related transcription factor 2 (RUNX2), ALP, bone morphogenetic protein 2 (BMP2), and collagen type I (COL1) transcription were markedly upregulated in EfnB2-hDPSCs relative to Vector-hDPSCs on days 7, 14, and 21, and osteocalcin (OCN) transcription was upregulated on day 7 (Fig. 3c–e). We also detected mRNA expression of odontogenic markers, including dentin sialophosphoprotein (DSPP) and dentin matrix protein 1 (DMP1), which was not affected by ephrinB2 overexpression (Fig. 3c–e).

### EphrinB2 overexpression promoted osteogenic differentiation of hDPSCs partially via enhancement of ephrinB2-mediated reverse signaling

**Fig. 4:**
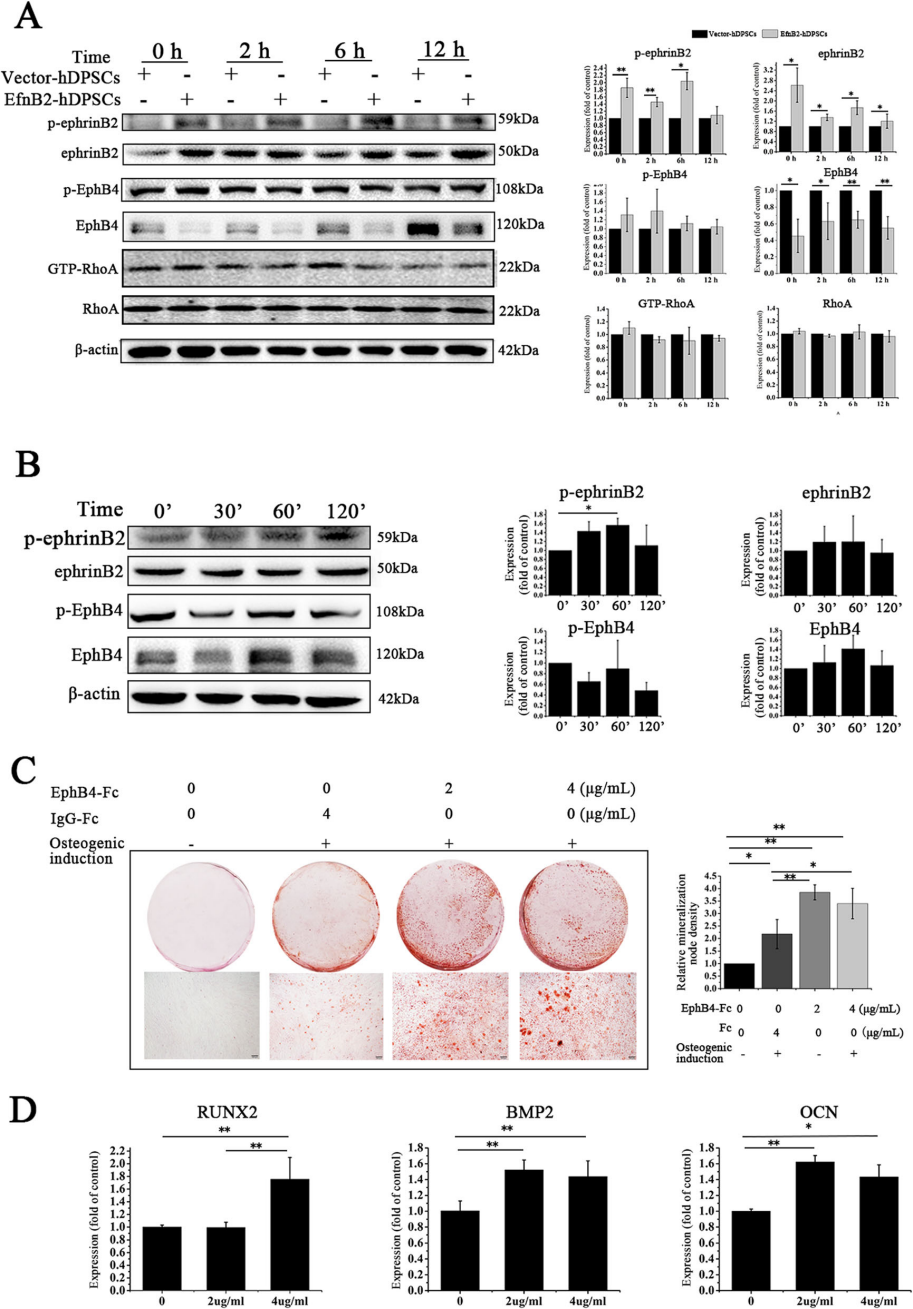
EphrinB2 overexpression promoted osteogenic differentiation of hDPSCs partially via enhancement of ephrinB2-mediated reverse signaling. **a** Overexpression of ephrinB2 elevated p-ephrinB2 but did not affect p-EphB4 and RhoA activity in hDPSCs during osteogenic differentiation. **b** Western blot analysis confirmed that ephrinB2 reverse signaling was activated by 1 μg/ml EphB4-Fc. **c** Alizarin Red S staining of hDPSCs treated with EphB4-Fc at different concentrations (0, 2, and 4 μg/ml) in osteogenic medium for 14 days. Scale bar = 200 μm. **d** Transcription of RUNX2, BMP2, and OCN in EphB4-Fc-treated hDPSCs on day 7 of osteogenic induction. Data are shown as mean ± SD. Assays were repeated three times. *p < 0.05 and **p < 0.01

The ephrinB2-EphB4 interaction plays a critical role in osteogenesis. Therefore, we hypothesized that ephrinB2 overexpression might elevate the ephrinB2-EphB4 interaction, thereby stimulating osteogenic differentiation of hDPSCs. First, we compared p-ephrinB2 and p-EphB4 in EfnB2-hDPSCs with those in Vector-hDPSCs. Western blot analysis showed that the p-ephrinB2 level was higher in EfnB2-hDPSCs at 0, 2, and 6 h, along with a continuously higher level of ephrinB2 in EfnB2-DPSCs (Fig. 4a). However, EphB4 mRNA and protein expression levels were downregulated in EfnB2-hDPSCs, which was surprising (Fig. 4a and Additional file 1: Figure S1A, B). May be due to downregulation of EphB4, p-EphB4 was not elevated in EfnB2-hDPSCs (Fig. 4a). We also detected the protein expression level of other EphB receptors, EphB1 and EphB2, which was not affected by ephrinB2 overexpression (Additional file 1: Figure S1C). These results indicated that ephrinB2 overexpression in hDPSCs enhanced ephrinB2-mediated reverse signaling instead of EphB4-mediated forward signaling.

To further test the role of EphB4-mediated forward in the osteogenic differerentiation of ephrinB2-overexpression hDPSCs, we detected active GTP-RhoA that is the downstream target of EphB4-mediated forward signaling in the process of osteogenesis. Results showed that active GTP-RhoA expression was not affected by ephrinB2 overexpression.

Next, the stimulating effect of ephrinB2-mediated reverse signaling on osteogenic differentiation of hDPSCs was explored. EphB4-Fc only interacts with the ephrinB2 ligand; therefore, it is commonly used to selectively activate ephrinB2-mediated reverse signaling. In our study, western blot analysis showed that ephrinB2-mediated reverse signaling was activated by EphB4-Fc (Fig. 4b). hDPSCs were then treated with 2 or 4 μg/ml EphB4-Fc, and their osteogenic differentiation was tested. Results revealed that there were more calcium deposits in EphB4-Fc treated hDPSCs compared with that in IgG-Fc-treated hDPSCs and uninduced hDPSCs (Fig. 4c). In addition, EphB4-Fc treatment also increased RUNX2, BMP2, and OCN transcription (Fig. 4e).

### Culture, characterization, and transfection of cDPSCs and encapsulation of cDPSCs with PuraMatrix

**Fig. 5:**
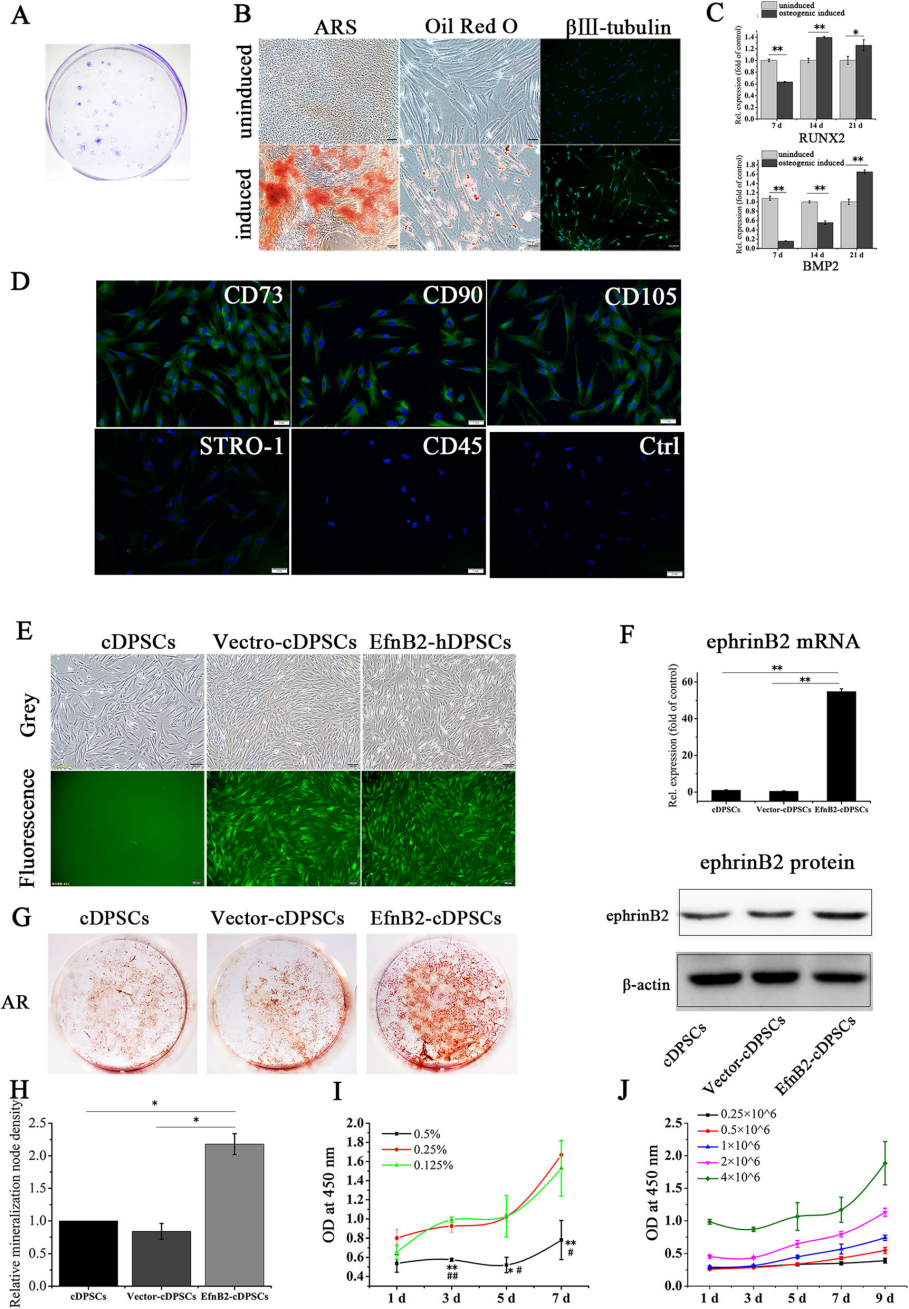
Culture, characterization, and transfection of cDPSCs and proliferation of cDPSCs in PuraMatrix. **a** Cell colonies stained with crystal violet. **b, c** Verification of osteogenic, adipogenic, and neurogenic differentiation capabilities of cDPSCs. Scale bar of left and right images, 100 μm; scale bar of middle images, 50 μm. *p < 0.05 and **p < 0.01. **d** Stem cell markers of cDPSCs. Scale bar = 1 mm. **e, f** Verification of green fluorescence expression and ephrinB2 upregulation in transfected cDPSCs. Scale bar = 100 μm. **p < 0.01. **g** Alizarin Red S staining of cDPSCs, Vector-cDPSCs (ephrinB2 overexpression control), and EfnB2-cDPSCs (ephrinB2 overexpression) on day 24 of osteogenesis. **h** Alizarin Red S staining intensity was quantified with ImageJ. *p < 0.05. **i** Proliferation of cDPSCs (1 × 10^6^ cells/ml) in 0.5%, 0.25%, and 0.125% PuraMatrix. *p < 0.05 and **p < 0.01 vs. 0.25% PuraMatrix; # *p* < 0.05 and ## *p* < 0.01 vs. 0.125% PuraMatrix. **j** Proliferation of cDPSCs at different cell densities (0.25, 0.5, 1, 2, or 4 × 10^6^ cells/ml) in 0.25% PuraMatrix. Data are shown as mean ± SD. Assays were repeated three times

The studies above demonstrated that ephrinB2-overexpressing DPSCs possessed higher osteogenic potential in vitro. To determine the effect of ephrinB2-overexpressing DPSCs in vivo, a beagle dog experiment was performed. cDPSCs were isolated from canine anterior teeth, and their colony-forming abilities and multi-differentiation potentials were demonstrated (Fig. 5a–c). Immunofluorescence analysis revealed that they were positive for CD73, CD90, CD105, and STRO-1, and negative for CD45 (Fig. 5d). cDPSCs were then infected with EfnB2 lentiviral vectors, and the high transfection efficiency was confirmed by green fluorescence expression (Fig. 5e) and ephrinB2 upregulation (Fig. 5f). Similar to EfnB2-hDPSCs, ephrinB2-overexpressing cDPSCs (EfnB2-cDPSCs) possessed elevated osteogenic potential relative to uninfected cDPSCs and control vector-infected cDPSCs (Vector-cDPSCs), as indicated by Alizarin Red S staining (Fig. 5g).

To effectively transfer cells into the defective regions, cDPSCs were encapsulated in PuraMatrix. Cell proliferation assays revealed that cDPSCs proliferated more slowly in 0.5% PuraMatrix (Fig. 5h). PuraMatrix at 0.125% dilution is very loose and easily disrupted. Therefore, 0.25% was the appropriate PuraMatrix concentration. The proliferation of cDPSCs at different densities in 0.25% PuraMatrix revealed that 4 × 10^6^ cells/ml is the optimal cell density, which could guarantee sufficient cell numbers and satisfactory proliferation (Fig. 5i).

### EphrinB2 overexpression cDPSCs promoted defect repair in a canine alveolar bone defect model

**Fig. 6:**
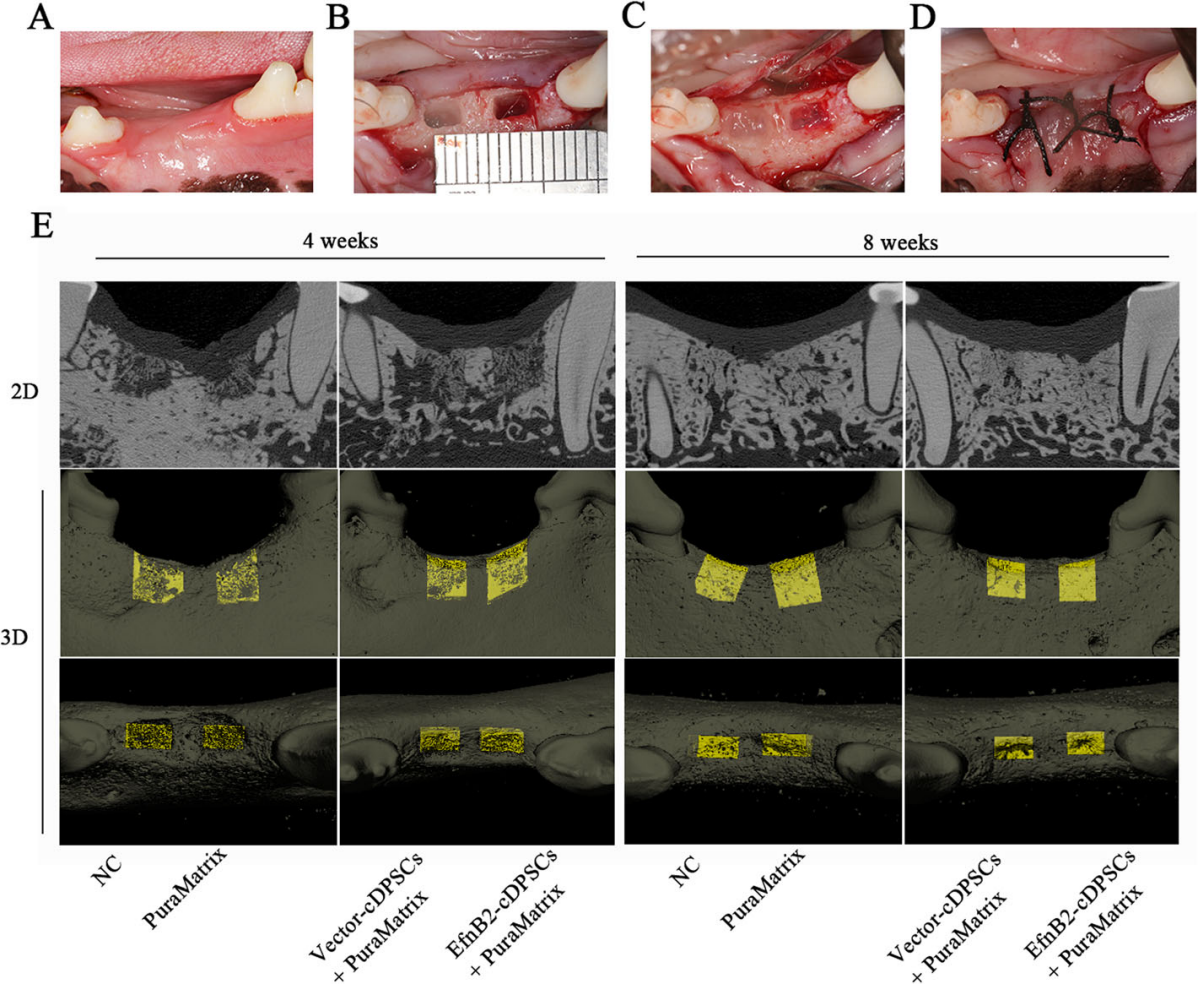
Transplantation of ephrinB2 overexpression cDPSCs into bone defects and evaluation of bone regeneration. **a–d** Construction of alveolar bone defect models and transplantation of ephrinB2 overexpression cDPSCs. **e** 2D and 3D micro-CT images of new bone formation in the defect areas at the 4th and 8th week

**Fig. 7:**
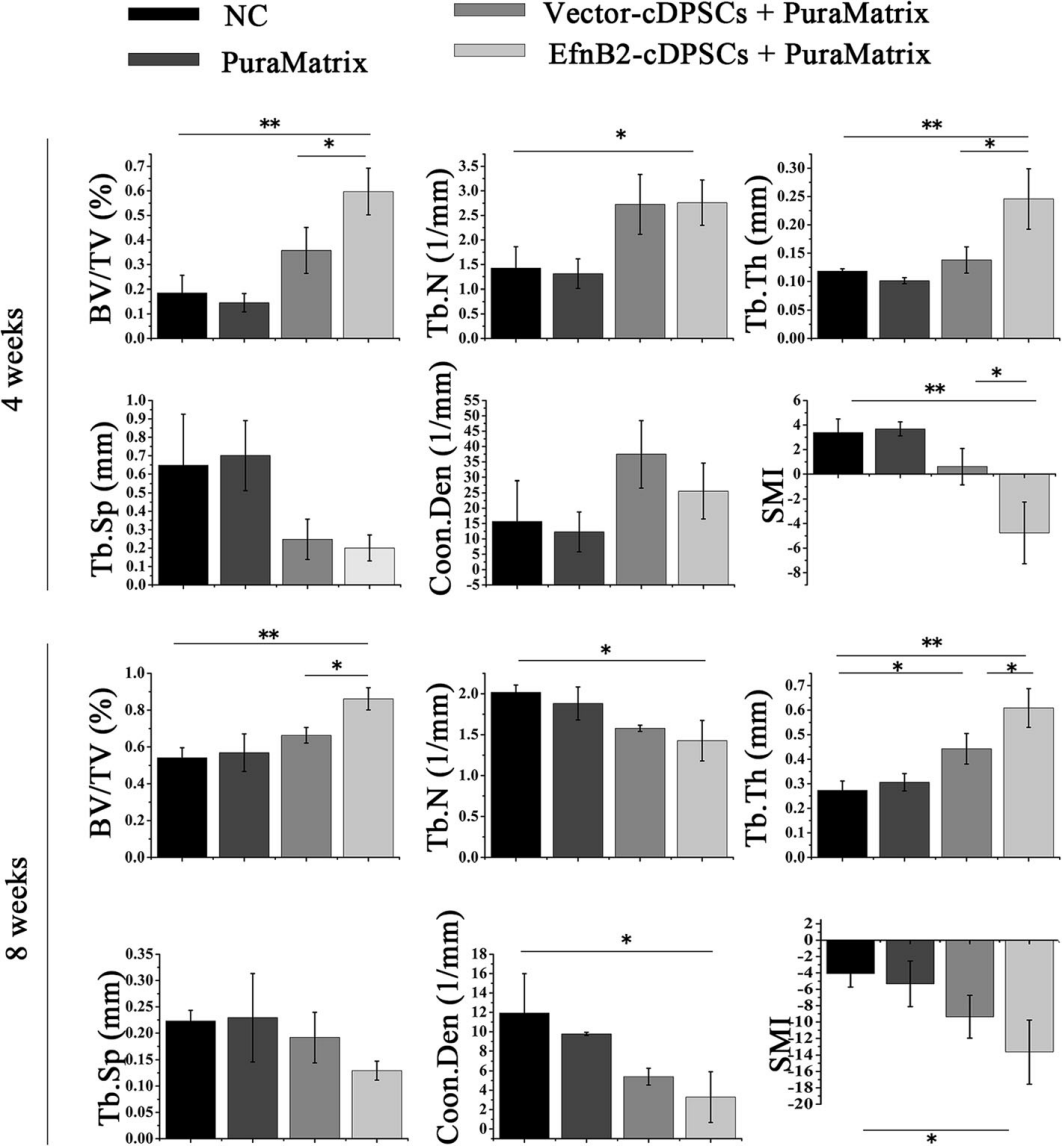
Quantitative analysis of new bone volume and trabecular parameters at the 4th and 8th week. Data are shown as mean ± SD (*n* = 3). *p < 0.05 and **p < 0.01. BV/TV = trabecular bone volume per tissue volume; Tb.N = trabecular number; Tb.Th = trabecular thickness; Tb.Sp = trabecular spacing; Conn. Den = connectivity density; SMI = structure model index

A canine alveolar bone defect model was constructed (Fig. 6a–d) and the effect of ephrinB2 overexpression cDPSCs on bone damage repair was observed at the 4th and 8th week from the NC, PuraMatrix, Vector-cDPSCs + PuraMatrix, and EfnB2-cDPSCs + PuraMatrix groups. The 2-D images of representative sagittal slices and 3-D reconstruction images showed that the new mineralized tissues were denser with less lacunas and cavities in the EfnB2-cDPSCs + PuraMatrix group compared with the other groups (Fig. 6e). At the 4th week after the operation, analysis revealed that the BV/TV of the EfnB2-cDPSCs + PuraMatrix group was 1.67-fold higher and 3.22-fold higher than that of the Vector-cDPSCs + PuraMatrix group and NC group, respectively. In addition, the EfnB2-cDPSCs + PuraMatrix group had a higher Tb.Th and lower SMI compared with the other groups (Fig. 7). At the 8th week, the EfnB2-cDPSCs + PuraMatrix group still had the highest BV/TV (1.30-fold higher than the Vector-cDPSCs + PuraMatrix group; 1.59-fold higher than the NC group). EfnB2-cDPSCs still resulted in a higher Tb.Th compared to Vector-cDPSCs and NC (Fig. 7). Micro-CT analysis demonstrated that there were more and denser mineralized tissues in the EfnB2-cDPSCs + PuraMatrix group compared with the other groups.

## Discussion

Successful and sufficient osteogenesis is essential when DPSCs are applied to bone tissue engineering and regeneration. While studies have proven DPSCs enhancing bone regeneration in vitro and in vivo, large volume bone formation by DPSC-based therapy has not yet achieved [12]. It is urgent to find a way to upregulate DPSC osteogenic capacity for broader clinical application. To the best of our knowledge, this is the first study providing evidence that ephrinB2 overexpression offers a novel strategy to enhance osteogenic capacity of DPSCs, thereby promoting DPSC-based bone regeneration.

EphrinB2 expressed on osteoclasts or exogenous ephrinB2-Fc have been verified to stimulate osteogenesis of osteoblasts and BMSCs, mainly via interaction with the EphB4 receptor [30, 37–40]. Endogenous ephrinB2 expressed on osteoblasts also plays pivotal roles in bone formation and mineralization [33, 34, 41, 42]. However, the expression and role of endogenous ephrinB2 in DPSCs under osteogenic differentiation has not been investigated yet. In this study, we observed that ephrinB2, p-ephrinB2 and its receptor EphB4 in hDPSCs were upregulated after osteogenic induction of hDPSCs, which is consistent with a previous report [36]. Meanwhile, p-EphB4 expression was steady during this osteogenesis process. These results proved that ephrinB2 and its receptor EphB4 were co-expressed on hDPSCs and suggested the involvement of ephrinB2 in osteogenic differentiation of hDPSCs. To elucidate the osteogenic effect of endogenous ephrinB2, we upregulated ephrinB2 expression in hDPSCs via lentiviral EfnB2 transfection, and the results revealed that ephrinB2 overexpression significantly increased mineral deposition and upregulated osteogenic genes instead of odontogenic genes in hDPSCs.

Previous studies have indicated that exogenous ephrinB2-Fc enhances osteoblast differentiation via stimulating EphB4-mediated forward signaling [30], and endogenous ephrinB2 might act in a paracrine or autocrine manner on EphB4 in osteoblasts to promote osteogenesis [33]. Thus, we speculated that ephrinB2 overexpressed on hDPSCs might bind to and activate the EphB4 receptor, thereby accelerating osteogenesis of hDPSCs. Contrary to this, there was no upregulation of phosphorylation of EphB4 and EphB4 expression decreased at both mRNA and protein levels in ephrinB2-overexpressing hDPSCs. The negative feedback regulation of the Eph receptor expression by the ephrin ligand has also been reported by previous studies, which revealed that lack of ephrinB1 expression led to post-transcriptional upregulation of the EphB receptor expression through relief of endocytosis and degradation [43]. Furthermore, we found that GTP-RhoA, which is a downstream target of EphB4-mediated forward signaling in the process of osteogenesis, was not affected by ephrinB2 overexpression. These results excluded the contribution of EphB4-mediated forward signaling in the osteogenesis of ephrinB2-overexpressing hDPSCs.

In this study, we identified elevated activation of ephrinB2 reverse signaling in ephrinB2 overexpression hDPSCs. Although the role of ephrinB2 reverse signaling in osteogenesis is unclear, ephrinB1-mediated reverse signaling has been proven to stimulate osteogenic differentiation of BMSCs and osteoblasts by influencing PDZ binding motif (TAZ) transactivation [44, 45]. To further assess the effect of ephrinB2-mediated reverse signaling on osteogenesis, we used EphB4-Fc to specifically activate ephrinB2 signaling, since EphB4 only binds with the ephrinB2 ligand instead of other ligands [46, 47]. We found EphB4-Fc treatment resulted in more calcium nodule formation in hDPSCs together with an increase in osteogenic gene transcription, which indicated the contribution of ephrinB2-mediated reverse signaling to osteogenic differentiation of hDPSCs. Taken together, these findings suggested that ephrinB2 overexpression promoted osteogenic differentiation of hDPSCs partially through activation of ephrinB2-mediated reverse signaling.

Although the ephrinB2/EphB4 interaction is mostly studied, EphB1 and EphB2 have also been reported as likely candidate receptors for ephrinB2 in calvarial bone formation [31]. We detected EphB1 and EphB2 expression patterns in ephrinB2-overexpressing hDPSCs and found that EphB1 and EphB2 expression were not affected by ephrinB2 overexpression. This may partially account for the phosphorylation of ephrinB2 in the absence of EphB4. However, the osteogenic function of EphB1 and EphB2 in DPSCs has not been reported before and whether ephrinB2 overexpression stimulated osteogenesis partially through EphB1 or EphB2 mediated forward signaling requires further research.

To explore the impact of ephrinB2 overexpression DPSCs on bone regeneration in vivo, a canine alveolar bone defect model was established and EfnB2-modified cDPSCs encapsulated with PuraMatrix hydrogel were transplanted into defects. We found that ephrinB2 overexpression cDPSCs accelerated bone defect repair, as indicated by an increased BV/TV and Tb.Th. It has been reported that gene-modified stem cells, such as osteoprotegerin (OPG)-modified periodontal ligament stem cells, promote bone defect repair in vivo [48, 49]. There were various methods to evaluate new bone formation, such as radiography, micro-CT, and histological examination. We observed greater and denser new mineralized tissues radiographically by ephrinB2 overexpression cDPSCs. Further studies are needed to histologically verify bone matrix formation instead of dentin matrix formation and vascularization of the newborn tissues [50].

## Conclusions

In conclusion, this study demonstrated that ephrinB2 was upregulated in hDPSCs under osteogenic differentiation. EphrinB2 overexpression enhanced osteogenic potential of hDPSCs partially via ephrinB2-mediated reverse signaling in vitro, and ephrinB2-modified cDPSCs accelerated bone regeneration in a canine bone defect model. Thus, EphrinB2 signaling might be a potential target to enhance osteogenesis of DPSCs.

## Supplementary information

Additional file 1: **Figure S1.** Expression patterns of EphB1, EphB2, and EphB4 in ephrinB2-overexpressing hDPSCs. (A) EphB4 mRNA expression level was lower in ephrinB2-overexpressing hDPSCs on days 0 and 7 of osteogenic induction. (B) EphB4 protein expression level was lower in ephrinB2-overexpressing hDPSCs on days 0 and 7 of osteogenic induction. (C) Overexpression of ephrinB2 did not affect EphB1 and EphB2 protein expression levels. Data are shown as mean ± SD. Assays were repeated three times. *p < 0.05 and **p < 0.01 (TIF 10113 kb)

## Abbreviations

ALP: Alkaline phosphatase
bFGF: Basic fibroblast growth factor
BMP2: Bone morphogenetic protein 2
BMSCs: Bone mesenchymal stem cells
BV/TV: Trabecular bone volume per tissue volume
CCK-8: Cell Counting Kit-8
cDPSCs: Canine dental pulp stem cells
COL1: Collagen type I
Conn-Den: Connectivity density
DMP1: Dentin matrix protein 1
DPSCs: Dental pulp stem cells
DSPP: Dentin sialophosphoprotein
EGF: Epidermal growth factor
FBS: Fetal bovine serum
hDPSCs: Human dental pulp stem cells
MSCs: Mesenchymal stem cells
OCN: Osteocalcin
OPG: Osteoprotegerin
qRT-PCR: Quantitative real-time reverse transcription-polymerase chain reaction
RUNX2: Runt-related transcription factor 2
SAPs: Self-assembling peptides
SMI: Structure model index
Tb.N: Trabecular number
Tb.Sp: Trabecular spacing
Tb.Th: Trabecular thickness
α-MEM: α-Minimum essential medium
