# Supplementary figures and images for: EphrinB2 overexpression enhances osteogenic differentiation of dental pulp stem cells partially through ephrinB2-mediated reverse signaling
Source: Stem Cell Res Ther. 2020 Jan 29;11:40. doi: 10.1186/s13287-019-1540-2 (PMC6990579; doi:10.1186/s13287-019-1540-2)

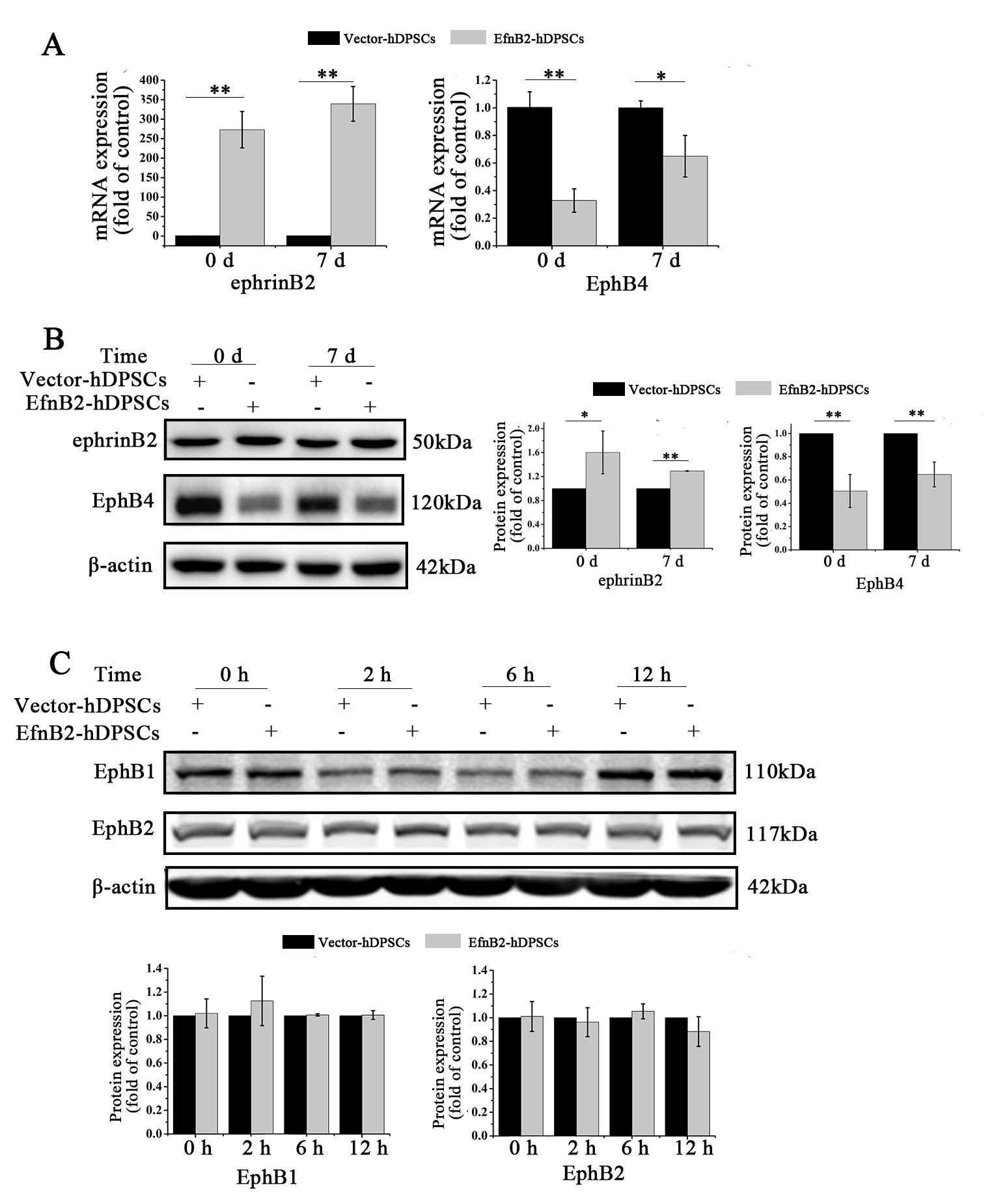

Supplement: Supplementary file 1 — Figure S1. Expression patterns of EphB1, EphB2, and EphB4 in ephrinB2-overexpressing hDPSCs. (A) EphB4 mRNA expression level was lower in ephrinB2-overexpressing hDPSCs on days 0 and 7 of osteogenic induction. (B) EphB4 protein expression level was lower in ephrinB2-overexpressing hDPSCs on days 0 and 7 of osteogenic induction. (C) Overexpression of ephrinB2 did not affect EphB1 and EphB2 protein expression levels. Data are shown as mean ± SD. Assays were repeated three times. *p < 0.05 and **p < 0.01 (TIF 10113 kb) [file 13287_2019_1540_MOESM1_ESM.tif]
